# Coating formulation change leads to inferior performance of long-lasting insecticidal nets in Papua New Guinea

**DOI:** 10.1186/s12936-022-04392-3

**Published:** 2022-11-24

**Authors:** Nakei Bubun, Evodia Anetul, Melanie Koinari, Timothy W. Freeman, Stephan Karl

**Affiliations:** 1 Vector-Borne Diseases Unit, Papua New Guinea Institute of Medical Research, Madang, Madang Province Papua New Guinea; 2 Australian Institute of Tropical Health and Medicine, James Cook University, Smithfield, QLD Australia; 3 Rotarians Against Malaria Papua New Guinea, Port Moresby, National Capital District, Papua New Guinea

## Abstract

**Background:**

Long-lasting insecticidal nets (LLINs) play a key role in reducing malaria transmission in endemic countries. In a previous study, the authors demonstrated a substantial decrease in the bioefficacy of LLINs for malaria prevention delivered to Papua New Guinea (PNG) between 2013 and 2019. This coincided with a rise in malaria cases in the country. The present study was aimed at determining the underlying cause of the reduced bioefficacy observed in these LLINs. The main hypothesis was that a change in the coating formulation of the respective LLIN product was responsible, and had led to significantly altered product properties and performance.

**Methods:**

A set of PermaNet^®^ 2.0 LLIN samples (n = 12) manufactured between 2007 and 2019 was subjected to combustion ion chromatography in order to understand the chemistry of the LLIN polymer coating formulation. In addition, World Health Organization (WHO) LLIN standard wash tests and cone bioassays were conducted to further characterize the change in product performance that occurred between 2012 and 2013.

**Results:**

High polymer fluorine content (average 3.2 g/kg) was measured in PermaNet^®^ 2.0 manufactured up to 2012, whereas nets which were manufactured after 2012 contained very little polymer fluorine (average 0.04 g/kg) indicating a coating formulation change from a fluorocarbon (FC)-based to a non-FC-based formulation. The coating formulation change as part of the manufacturing process thus resulted in a significant reduction in bioefficacy. In addition, the manufacturing change affected wash resistance leading to a faster reduction in 24 h mosquito mortality in the non-FC-coated product with consecutive washes.

**Conclusion:**

A change in coating formulation of PermaNet^®^ 2.0 resulted in reduced product performance in PNG. Post-2012 PermaNet^®^ 2.0 LLINs should not be considered to be the same product as PermaNet^®^ 2.0 LLINs produced prior to and in 2012. Coating formulation changes should be validated to not impact LLIN product performance.

**Supplementary Information:**

The online version contains supplementary material available at 10.1186/s12936-022-04392-3.

## Background

Long-lasting insecticidal nets (LLINs) are the most important vector control tool against malaria [1]. No other method is considered to have prevented more cases and saved more lives [2]. LLINs protect by providing a physical barrier between the user and potentially infectious mosquitoes. Importantly, they also afford community protection through efficiently killing mosquitoes that come into contact with the insecticide-treated surfaces [3]. Billions of LLINs have been distributed with public donor funding. In order to be eligible for donor procurement LLIN products must pass a WHO prequalification process [4].

By conducting post-delivery bioefficacy testing of LLINs in Papua New Guinea (PNG), Vinit et al*.* [5] recently identified a substantial reduction in the ability of a leading LLIN product to kill susceptible *Anopheles farauti* colony mosquitoes. Specifically, bioefficacy was observed to be reduced for PermaNet^®^ 2.0 LLINs with manufacturing dates post-2012 [5]. This was surprising as PermaNet^®^ 2.0 had been the only WHO prequalified LLIN product distributed in PNG from 2007 until 2019 and thus, consistent product performance had been expected. PNG is the country with the highest malaria transmission outside of Africa. However, the *Anopheles* populations in PNG remain phenotypically susceptible to pyrethroids and, therefore, next generation LLIN products are currently not being supplied to PNG [6]. The distribution of these significantly less potent LLINs in PNG starting in 2013 coincided with a resurgence of malaria in the country, leading to the hypothesis that the decreased community-level protection afforded by these inferior LLINs had contributed to the observed increase in malaria case numbers [5].

It has remained unclear what had caused the reduction in performance of the LLINs in PNG. Hypotheses to explain these observations included inappropriate transport, and short-term storage conditions of the new and unused nets after 2013, such as exposure to elevated temperatures in shipping containers [7]. This was unlikely, as the overall insecticide content in the tested LLINs from all years (2007 to 2019) was similar, i.e., container storage had not resulted in a rapid breakdown of the insecticide [4]. Also, older nets that had been stored for much longer under tropical conditions and exhibited 100% 24 h mosquito kill rate had, on average, slightly lower insecticide content. This can most likely be attributed to the expected natural decay over many years of storage [4]. In addition, it was shown that short-term heating of the LLINs in question increased their potency to kill mosquitoes (rather than to decrease it), which could potentially be explained by heat-facilitated migration of the insecticide from inside the LLINs’ polymer coating to the net surface [8, 9]. It was also suspected that the mosquito strain that had been used (a fully pyrethroid susceptible strain of *Anopheles farauti*) or technicalities related to conducting WHO cone bioassays at the PNG Institute of Medical Research were responsible for the observed inferior LLIN performance. These possibilities were ruled out categorically, by subsequent multi-centre trials with the same LLIN samples, showing that the observations from PNG were reproducible in an African ‘Good Laboratory Practice’- accredited facility [10].

The LLIN product that was distributed in PNG between 2007 and 2019 (PermaNet^®^ 2.0) is a polyester net with a polymer coating that contains the insecticide. While predelivery inspections had verified the total insecticide content of all nets to be within specifications [4], the formulation of the polymer coating may also influence insecticidal potency of a net [9]. This is because some coating technologies and formulations may result in a more effective presentation of the insecticide on the net surface where it comes into contact with mosquitoes, whereas other coatings may enclose the insecticide under a polymer layer, and restrict its bioavailability [9, 11].

Polymer coatings in the textile industry can be grouped into a few major classes, with a major distinction between fluorocarbon (FC)-based and non-FC-based coatings. Fluorocarbons are organic compounds consisting of perfluorinated carbon chains. Since 1990s, FC-based coatings dominated the textile industry due to their unique properties such as repellency to water, oil, stain and soil [12]. Non-FC-based coatings are typically acrylates, polyurethanes or mixtures thereof [13, 14]. These are cheaper and considered more environmentally friendly.

The industrial standard to distinguish between FC-based and non-FC-based formulations is the detection of fluorine in the coating polymer, as it is only found in FC-based coatings. The reference method to measure total polymer fluorine content in textile samples is combustion ion chromatography. In the present study, PermaNet^®^ 2.0 LLINs manufactured before the observed bioefficacy shift (before 2013) and after the observed bioefficacy shift (2013–2019) were characterised using combustion ion chromatography and WHO wash resistance assays.

## Methods

### LLIN sampling

As described in Vinit et al. [5], unused LLINs manufactured in 2018 and 2019 were provided by Rotarians against Malaria (RAM) PNG from consignments dedicated to different PNG provinces, whereas unused LLINs manufactured in 2007–2017 were obtained from villages or provincial health authorities in various PNG provinces. All LLINs were still in original and unopened packaging. The full list and data can be found as supporting information to Vinit et al. [5].

Two samples from each of the following years, 2008, 2010, 2012, 2015, 2017, 2019 were randomly selected from the collected sample set for polymer fluorine analysis. The list of selected nets can be found in Additional file 1: Table S1. LLIN samples for the wash tests were randomly selected from the same batches as the nets used for chemical analysis. Details can be found in Additional file 1: Table S2.

### Combustion ion chromatography

To test the hypothesis of a major coating formulation change, we submitted a total of n = 12 LLINs for combustion ion chromatography conducted by an independent, globally recognized reference laboratory (SGS, Australia). Specifically, the tested samples were from LLINs also used in the original study conducted by Vinit et al*.* [5] from the following years: 2008 (n = 2), 2010 (n = 2), 2012 (n = 2), 2015 (n = 2), 2017 (n = 2) and 2019 (n = 2). Given that the change in the bioefficacy of the nets occurred after 2012, it was expected that the first 6 nets (2008–2012) were coated with one specific coating formulation, whereas the other 6 nets (2015–2019) would be coated with another coating formulation.

### LLIN washing procedure

LLINs were washed according to WHO guidelines [15]. Briefly, n = 7 net samples of 25 cm × 25 cm in size were cut from random positions of each whole net. Net samples were introduced individually into 1 L glass bottles (Duran, Sigma Aldrich) containing 500 mL tap water, with 2 g/L mild local soap (pH 10–11). The soap was added and fully dissolved just before washing. The bottles were placed into a water bath shaker (Julabo SW22, John Morris Group) set to a temperature of 30 °C and shaken for 10 min at 155 movements per minute [15]. The samples were then rinsed twice for 10 min with tap water using the same shaking conditions, dried at room temperature and stored in a laboratory incubator (Heratherm, IMH60, Thermofisher Scientific) at 30 °C in the dark between washes. In order to account for the potential effect of the local soap, we also conducted the wash assays using only water. A total of n = 2 LLINs from 2012 and n = 2 LLINs from 2019 were subjected to WHO wash assays.

### WHO cone bioassays

Cone bioassays were conducted after 0, 1, 3, 5, 10, 15, 20 and 25 washes as described previously [5], in adherence with WHO guidelines and with confirmed fully pyrethroid susceptible, 3–5 days old female *An. farauti* mosquitoes. Bioassays were always performed just before the next wash. Tests were conducted in ambient tropical environment (Madang, PNG, latitude 5° south), and temperature and humidity requirements were met in all assays included in the study. The number of mosquitoes per cone was n = 5 (4 cones were used per 25 × 25 cm net piece) and exposure time was 3 min.

All cone bioassays included positive and negative controls. LLINs manufactured in 2012 and with a known 100% 24 h mortality were used as positive controls and pieces of untreated netting were used as negative controls. After exposure to the LLINs, mosquitoes were gently transferred from the cones to cardboard holding cups screened with untreated netting and provided access to 10% sugar solution via a soaked piece of cotton wool placed on top of the netting. After 24 h, the number of dead mosquitoes in the holding cups was enumerated.

Results were excluded and tests repeated, if 24 h mortality in the negative control exceeded 10%. Test results were adjusted using ‘Abbott’s formula’ when negative control 24 h mortality was > 0% and ≤ 10%.

## Results

**Fig. 1:**
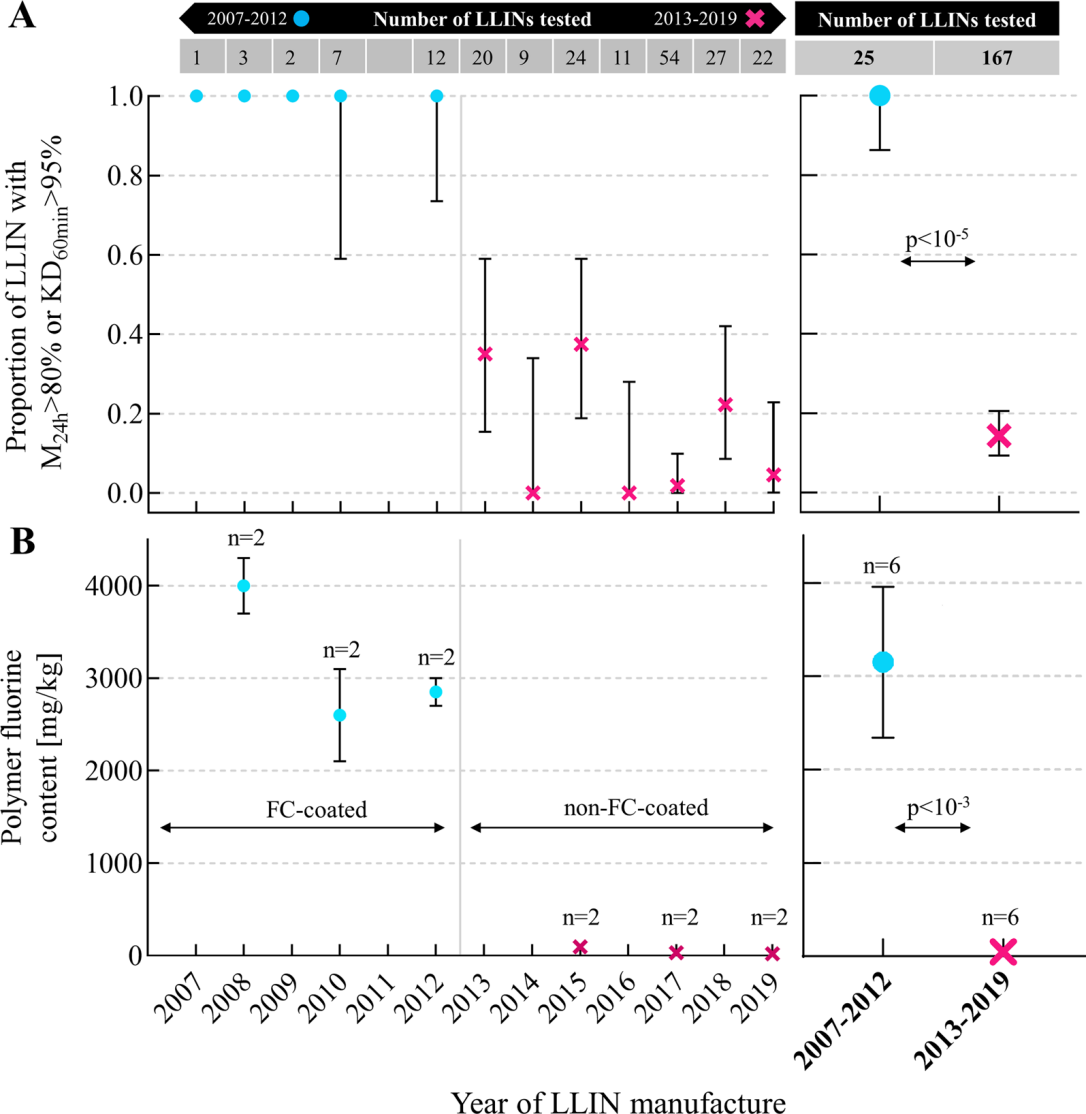
Change of coating formulation from FC-based to non-FC-based in PermaNet^®^ 2.0 and corresponding decreased bioefficacy. **A** Decreased bioefficacy of PermaNet^®^ 2.0 as presented in the original study [5], depicting the proportion of new and unused LLIN exhibiting ≥ 95% 60 min knockdown (KD_60min_) or ≥ 80% adjusted 24 h mortality (M_24h_) grouped by year of LLIN manufacture. Data is shown for individual years of manufacture on the left side and grouped by years 2007–2012 vs. years 2013–2019 on the right side. Data are presented as mean proportions and their exact 95% confidence intervals. **B** Corresponding polymer fluorine content as determined by combustion ion chromatography in the present study on n = 12 samples from the same LLIN batches as shown in A. Data are presented as means of n = 2 samples per year and the range. Data is shown for individual years of manufacture on the left side and grouped by years 2007–2012 (FC-coated) vs. years 2013–2019 (non-FC-coated) on the right side. Data for LLINs from before the coating formulation change (2007–2012) are shown as turquois circles. Data from LLINs manufactured after the coating formulation change (2013–2019) are shown as magenta crosses. X-axis applies to both panels A and B

Combustion ion chromatography analysis showed that the LLINs from before and including the year 2012 (n = 6) contained high amounts of fluorine, with an average fluorine content of 3.2 g/kg (range: 2.1–4.3 g/kg). The nets after 2012 (n = 6) contained only small trace amounts of fluorine (average 0.04 g/kg; range 0.005–0.12 g/kg) as shown in Fig. 1. As such, the coating formulation in the LLINs that killed mosquitoes very effectively (pre- or in 2012) and those that did not (post-2012) was fundamentally different. The analytical report by SGS is provided as Additional file 2.

The direct association between coating formulation and reduced bioefficacy in the n = 12 nets tested as part of this study was perfect, i.e. all non-FC-coated nets exhibited lower bioefficacy as compared to all FC-coated nets (p = 0.002; Additional file 1: Fig. S1).

**Fig. 2:**
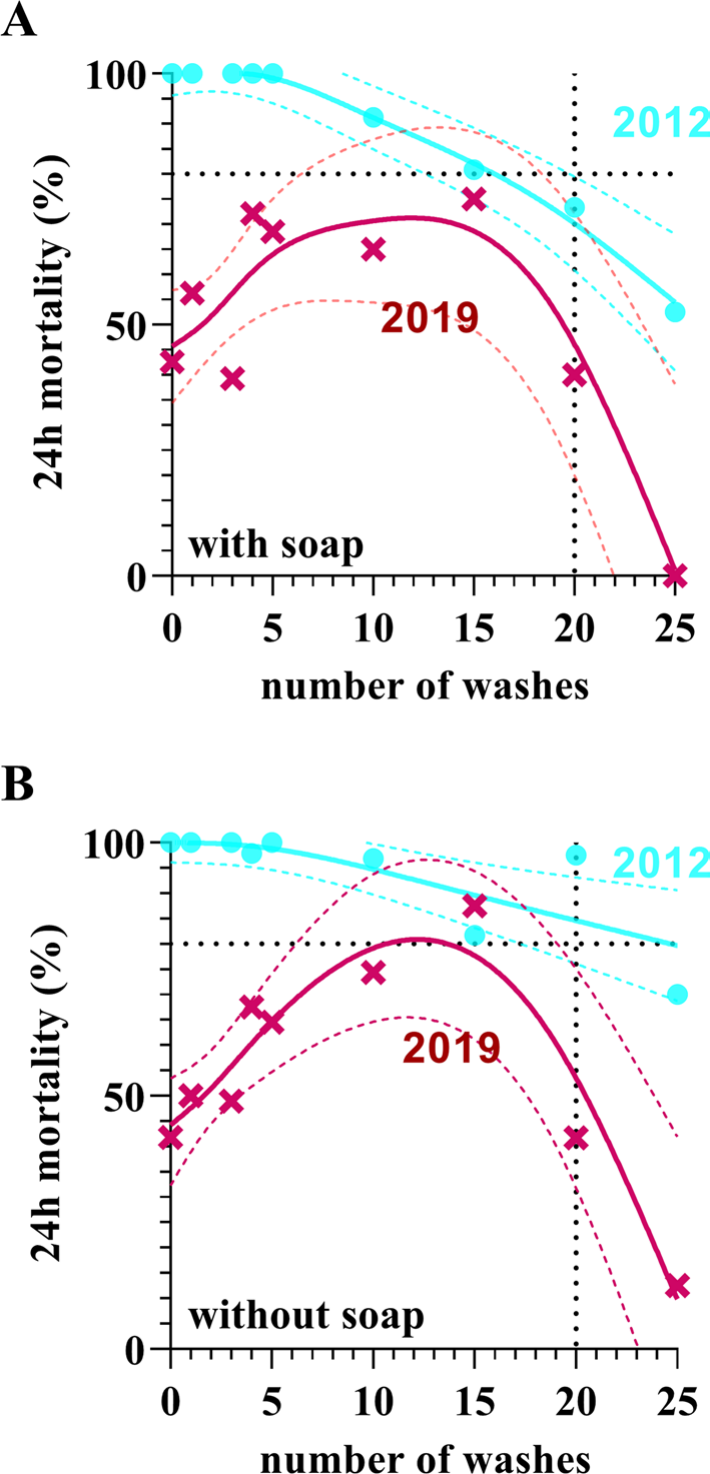
Standardized WHO cone bioassay results after washing. **A** shows cone bioassay data (24 h mortality) for PermaNet^®^ 2.0 nets LLINs from 2012 (n = 2 nets, 7 pieces per net) and LLINs from 2019 (n = 2, 7 pieces per net) washed according to WHO guidelines using a local soap. **B** To better understand the effect of the soap, we also conducted the same wash tests on the nets with water only, with no soap added at all. In both panels, data for LLINs from 2012 are shown as turquois circles and data from LLINs manufactured in 2019 are shown as magenta crosses. Data are presented as means (symbols). Also included are the means (bold lines) and the 95% confidence envelope of generalized additive model fits (dashed lines) to the raw data. The dotted lines indicate WHO thresholds of n = 20 washes and 80% mortality

The results from the WHO wash assays of the two product types are presented in Fig. 2. The analyses show that post-2012 PermaNet^®^ 2.0 LLINs (i.e., non-FC-coated LLINs) distributed in PNG performed poorly over their entire lifespan (up to 25 washes).

While FC-coated samples withstood up to 20 washes with an approx. 80% 24 h mosquito mortality, the non-FC-coated nets rarely reached 80% 24 h mortality at any number of washes, including 0 washes. With the non-FC-coated product, we also observed a slight increase in 24 h mortality after the first few washes, indicating wash-off of coating leading on an intermittently increased surface concentration of deltamethrin. The 24 h mortality in the non-FC-coated LLINs then reached a plateau around 5–10 washes (with soap) and 10–15 washes (without soap), before a steep decrease towards 0% bioefficacy after 25 washes.

## Discussion

The present data indicate that PermaNet^®^ 2.0 LLINs were produced with a FC-based coating formulation up to the year 2012. PermaNet^®^ 2.0 LLINs produced thereafter were manufactured using a non-FC-based coating formulation. Furthermore, the data show that FC-coated PermaNet^®^ 2.0 nets performed better than non-FC-coated PermaNet^®^ 2.0 nets in killing pyrethroid susceptible *An. farauti* mosquitoes in PNG when new, used in the field and also after 25 washes [5]. This was further confirmed in independent repetitions of the bioassays with different mosquito colonies [5, 10]. Despite this, both types of PermaNet^®^ 2.0 (FC-coated and non-FC-coated) contained similar amounts of deltamethrin, meaning that bioefficacy was affected by the coating formulation change only, leading to a completely altered LLIN product with a significantly inferior ability to kill susceptible *An. farauti* and *Anopheles gambiae* (Ifakara) colony mosquitoes [5, 10]. During the coating process, a polymer film forms around the polyester fibres and the insecticide crystals are bound in the polymer coating. Thermal curing enables more insecticide at the surface of a new net [9]. Polymer coatings are hydrophobic and this can reduce the insecticide loss during washing [16]. FC-based coating provides the fabric with an exceptional durability to water and oil repellency, that may be superior to FC-free finishes [13]. This is due to the electronegativity of the fluorine atoms and the orientation of the perfluorinated chains perpendicular to the textile fibres resulting in low surface energy and super-hydrophobicity [14, 17]. FC-free finishes commonly have a lower durability [13, 18]. The reason that PermaNet^®^ 2.0 LLINs manufactured up to 2012 (FC-coated) exhibit higher bioefficacy and wash resistance compared to post-2012 (non-FC-coated) is thus likely due to the superior durability of FC-based coating and a more efficient presentation of the deltamethrin on the net surface in the FC-coated product. The exact physico-chemical properties underlying the different performance of these different products with the same label should be further investigated. Also, FC-coated PermaNet^®^ 2.0 LLINs retained their bioefficacy up to 20 washes with an 80% 24 h mosquito mortality compared to < 50% in the non-FC-coated LLINs (Fig. 2). This could also be explained by the superior hydrophobicity and durability of the FC-based coating. For example, a previous study has shown that most of the original coating grains remain on FC-coated polyethylene terephathalate (PET) fabrics after washing and wearing tests [19]. While the evidence for the association of the coating formulation change and the related reduction in 24 h mortality in WHO cone bioassays is conclusive, the possibility that other changes to the product were made at the same time, which may also have had an effect on product performance, cannot be excluded. In addition, it may be that the new PermaNet^®^ 2.0 product with the altered coating formulation became more prone to potentially detrimental storage and transport conditions although there is currently no peer-reviewed evidence to support that transport and storage detrimentally affect LLIN performance at all. The results presented here provide important insights into how different coating formulations can affect the performance of LLINs. Further detailed studies are needed to optimize LLIN coatings not only for cost but also for performance.

It is difficult to determine if the reduced mosquito mortality as a consequence of the coating formulation change has also inadvertently been observed in other studies. Since PermaNet^®^ 2.0 has been in the market for a long-time, studies evaluating its performance are now less frequent. In addition, pyrethroid resistance has spread much further in the last decade, after the manufacturing change, masking performance issues such as that described here, when wild mosquito populations are used. Other studies also often do not provide manufacturing dates and it is thus unclear when tested PermaNet^®^ 2.0 products were manufactured. The origin of the tested samples (e.g., directly received from the manufacturer versus from a donor-procured consignment) may also play a role.

Notwithstanding this, there is a substantial body of literature demonstrating that PermaNet^®^ 2.0 manufactured before 2013 exhibited consistent 100% 24 h mortality at baseline (unused/unwashed nets) in WHO cone bioassays. This consistent performance was observed with a wide range of susceptible mosquito strains and species including *An. gambiae, Anopheles stephensi, Anopheles albimanus, Anopheles culicifacies, Anopheles arabiensis, Aedes aegypti* and *Culex quinquefasciatus* from across various continents (Oceania, Asia, Africa and the Americas), as well as in mosquito colonies reared in European laboratories. A non-exhaustive list of these studies with confirmed pre-2013 PermaNet^®^ 2.0 nets includes the following studies (24 h mortality rates obtained with WHO cone bioassays are provided in parentheses): Katusele et al. (100%) [20]; Kilian et al. (100%) [21]; Castellanos et al. (100%) [22]; Graham et al. (100%) [23]; Kweka et al. (100%) [24]; Okia et al. (100%) [25]; Sreehari et al. (100%) [26]; Kayedi et al. (100%) [27]; Jarramillo et al. (100%) [28]; Dery et al. (100%) [29], and Sood et al. (100%) [30].

In contrast to this, there is a small but growing number of studies showing that new and unused PermaNet^®^ 2.0 nets with confirmed later (post-2012) manufacturing dates tested with pyrethroid susceptible mosquitoes in WHO cone bioassays have not been performing as consistently. This includes Vinit et al. (40%) [5]; Bagheri et al. (22%) [31], and Thiery et al. (85%) [32]. It is also noteworthy, how the 24 h mortality profile after washing in Bagheri et al. (Fig. 1 in [31]) closely resembles the wash data obtained from PermaNet^®^ 2.0 manufactured in 2019 shown in Fig. 2 of the present study. Additional studies that have not tested new nets at baseline but after use, such as Villalta et al.(16% after 6 months of use) [11] further strongly suggest that this performance issue is not restricted to PNG but is being seen in Asia, Africa and Central America.

It should also be noted that the mosquito species, even if fully pyrethroid susceptible *Anopheles* mosquitoes are used, can result in large differences in the effect size observed when testing 24 h mortality with WHO cone bioassays. For example in a blinded multicentre study conducted by the PNG Institute of Medical Research (using *An. farauti*) and the Ifakara Health Institute (IHI, using *An. gambiae*), 24 h mortality of post-2012 (non-FC-coated) PermaNet^®^ 2.0 was systematically lower at IHI [10]. As such, the difference in 24 h mortality observed when comparing nets from before and after the 2012/13 manufacturing change may strongly depend on the mosquito species used in the tests.

## Conclusions

In conclusion, the present study demonstrates that the coating formulation of LLINs is a crucial product attribute that determines how effectively these essential public health commodities kill malaria mosquitoes. As stated in a recent ‘Insecticide-treated Net (ITN) Product Review Report’, it is recognized by WHO Vector Control Product Prequalification that the *‘complexities of ITN formulations and manufacturing processes can have a significant impact on the intended performance of the product’* [33]. However, coating formulations are presently not assessed as part of predelivery inspections and are not identified in currently available product specification documents. Total insecticide concentration, as measured in predelivery inspections, only partially relates to actual LLIN performance. LLIN coatings thus need to be controlled just as rigorously as total insecticide concentration, and any change to coating formulations needs to be validated to not detrimentally affect insecticidal performance of the product.

PermaNet^®^ 2.0 was the most widely distributed LLIN product in the world at the time with 750 million nets distributed since 2002. Given that LLIN coating formulations have not been controlled or regulated since mass distributions began, it is likely that many other LLIN manufacturers have also changed coating formulations over the years. While it is not possible to exactly quantify the number of malaria cases that could have been averted if this particular manufacturing change had not been made, it is likely that it has contributed to the stalling success in malaria case reduction across the world, and especially in countries that have relied solely on PermaNet^®^ 2.0, like PNG. It is therefore crucial, that essential properties (like coating formulation chemistry) of all currently prequalified LLIN products are precisely defined and comprehensive product specifications are publicly available.

## Supplementary Information

**Additional file 1: Table S1.** PermaNet^®^ 2.0 samples randomly selected for combustion ion chromatography including their 24h mortality and total polymer fluorine content.** Table S2**. PermaNet^®^ 2.0 samples randomly selected for WHO wash-resistance tests including their average 24h mortality (7 samples) at 0 washes. **Figure S1**. Cone bioassay mortality observed in FC-coated (pre-2012) vs non-FC coated (post-2012) LLINs in the present study. The association between coating formulation and bioefficacy is perfect i.e., all non-PFC coated LLINs exhibited reduced bioefficacy as compared to the PFC-coated LLINs. The groups were compared using an unpaired, non-parametric significance test (Mann Whitney U test). The difference in the medians is 72% (p = 0.002).

**Additional file 2.** **Total fluorine analytical report provided by SGS Australia Pty Ltd.** The report details total fluorine content measurement results obtained from combustion ion chromatography for PermaNet ® 2.0 samples manufactured between 2008 and 2019.

## Data Availability

The data set for this study is available on request from the corresponding author.
