# Supplementary material for: Coating formulation change leads to inferior performance of long-lasting insecticidal nets in Papua New Guinea
Source: Malar J. 2022 Nov 24;21:349. doi: 10.1186/s12936-022-04392-3 (PMC9685832; doi:10.1186/s12936-022-04392-3)
Supplement: Supplementary file 1 — Additional file 1: Table S1. PermaNet® 2.0 samples randomly selected for combustion ion chromatography including their 24h mortality and total polymer fluorine content. Table S2. PermaNet® 2.0 samples randomly selected for WHO wash-resistance tests including their average 24h mortality (7 samples) at 0 washes. Figure S1. Cone bioassay mortality observed in FC-coated (pre-2012) vs non-FC coated (post-2012) LLINs in the present study. The association between coating formulation and bioefficacy is perfect i.e., all non-PFC coated LLINs exhibited reduced bioefficacy as compared to the PFC-coated LLINs. The groups were compared using an unpaired, non-parametric significance test (Mann Whitney U test). The difference in the medians is 72% (p = 0.002). [file 12936_2022_4392_MOESM1_ESM.docx]

**Supporting Information File 1**

**Table S1 PermaNet® 2.0 samples randomly selected for combustion ion chromatography including their 24h mortality and total polymer fluorine content.**

| **Net ID** | **Brand** | **Year of Man.** | **Mortality (%)** | **Fluorine content (mg/kg)** |
| --- | --- | --- | --- | --- |
| CHI 039 | PermaNet 2.0 | 2008 | 96 | 4300 |
| KAV 002 | PermaNet 2.0 | 2008 | 100 | 3700 |
| CHI 024 | PermaNet 2.0 | 2010 | 100 | 2100 |
| EHP 028 | PermaNet 2.0 | 2010 | 100 | 3100 |
| EHP 032 | PermaNet 2.0 | 2012 | 100 | 3000 |
| GUL 008 | PermaNet 2.0 | 2012 | 100 | 2700 |
| GUL 004 | PermaNet 2.0 | 2015 | 92 | 120 |
| ENB 003 | PermaNet 2.0 | 2015 | 36 | 68 |
| EHP 005 | PermaNet 2.0 | 2017 | 20 | 37 |
| CHI 022 | PermaNet 2.0 | 2017 | 16 | 24 |
| WP 003 | PermaNet 2.0 | 2019 | 16 | 35 |
| WP 004 | PermaNet 2.0 | 2019 | 40 | 5.2 |

**Table S2. PermaNet® 2.0 samples randomly selected for WHO wash-resistance tests including their average 24h mortality (7 samples) at 0 washes.**

| **Net ID** | **Brand** | **Year of Man.** | **Mortality (%)** |
| --- | --- | --- | --- |
| EHP 033 | PermaNet 2.0 | 2012 | 100 |
| KAV 005 | PermaNet 2.0 | 2012 | 100 |
| NIP 001 | PermaNet 2.0 | 2019 | 73 |
| EWNB 004 | PermaNet 2.0 | 2019 | 12 |

**Figure S1: Cone bioassay mortality observed in FC-coated (pre-2012) vs non-FC coated (post-2012) LLINs in the present study.** The association between coating formulation and bioefficacy is perfect i.e., all non-PFC coated LLINs exhibited reduced bioefficacy as compared to the PFC-coated LLINs. The groups were compared using an unpaired, non-parametric significance test (Mann Whitney U test). The difference in the medians is 72% (p=0.002).
