# Supplementary material for: Coating formulation change leads to inferior performance of long-lasting insecticidal nets in Papua New Guinea
Source: Malar J. 2022 Nov 24;21:349. doi: 10.1186/s12936-022-04392-3 (PMC9685832; doi:10.1186/s12936-022-04392-3)
Supplement: Supplementary file 2 — Additional file 2. Total fluorine analytical report provided by SGS Australia Pty Ltd. The report details total fluorine content measurement results obtained from combustion ion chromatography for PermaNet ® 2.0 samples manufactured between 2008 and 2019. [file 12936_2022_4392_MOESM2_ESM.pdf]

ANALYTICAL REPORT

Client:	Stephan Karl<br>**James Cook University**<br>88 Macgregor Rd<br>Smithfield QLD 4878
Your Reference:	Long Lasting Insecticidal Net for Total Fluorine Analysis
SGS Report Number:	SP035171
Date of Receipt of Samples:	09/05/2022
Sample Description:	12 net samples individually wrapped in aluminium foil

The samples were analysed in accordance with your instructions. The results and associated information are contained in the following pages of the report. Should you have any queries regarding this report please contact the undersigned.

Reported by: Dr Tracey Yeung

Date: 18/05/2022

Report authorised by: Dr Peter Novella

Date: 18/05/2022

This document is issued, on the Client's behalf, by the company under its General Conditions of Service available on request and accessible at http://www.sgs.com/terms_and_conditions.htm. The client's attention is drawn to the limitation of liability, indemnification and jurisdiction issues defined therein.

Any other holder of this document is advised that information contained hereon reflects the company's findings at the time of its intervention only and within the limits of client's instructions, if any. The company's sole responsibility is to its client and this document does not exonerate parties to a transaction from exercising all their rights and obligations under the transaction documents.

This test report shall not be reproduced except in full, without written approval of the laboratory.

Background:

SGS Sydney was requested by Stephan Karl of James Cook University to analyse 12 net samples for total fluorine. The samples were received on 09/05/2022 and logged in with the following laboratory reference numbers:

Table 1: Sample Information

SGS ID	Client ID	Year of Manufacture
SP035171-1	PermaNet 2.0<br>Long Lasting Insecticidal Net 1	2008
SP035171-2	PermaNet 2.0<br>Long Lasting Insecticidal Net 2	2008
SP035171-3	PermaNet 2.0<br>Long Lasting Insecticidal Net 3	2010
SP035171-4	PermaNet 2.0<br>Long Lasting Insecticidal Net 4	2010
SP035171-5	PermaNet 2.0<br>Long Lasting Insecticidal Net 5	2012
SP035171-6	PermaNet 2.0<br>Long Lasting Insecticidal Net 6	2012
SP035171-7	PermaNet 2.0<br>Long Lasting Insecticidal Net 7	2015
SP035171-8	PermaNet 2.0<br>Long Lasting Insecticidal Net 8	2015
SP035171-9	PermaNet 2.0<br>Long Lasting Insecticidal Net 9	2017
SP035171-10	PermaNet 2.0<br>Long Lasting Insecticidal Net 10	2017
SP035171-11	PermaNet 2.0<br>Long Lasting Insecticidal Net 11	2019
SP035171-12	PermaNet 2.0<br>Long Lasting Insecticidal Net 12	2019

Methods Used:

A portion of each sample was completely combusted in an oxygen bomb. The resulting combustion products were diluted with water (Type I) and the concentration of fluorine was analysed by ion chromatography.

Analytical Results:

Table 2: Total Fluorine Analysis Results

SGS ID	Fluorine	Units
SP035171-1	4300	mg/kg
SP035171-2	3700	mg/kg
SP035171-3	2100	mg/kg
SP035171-4	3100	mg/kg
SP035171-5	3000	mg/kg
SP035171-6	2700	mg/kg
SP035171-7	120	mg/kg
SP035171-8	68	mg/kg
SP035171-9	37	mg/kg
SP035171-10	24	mg/kg
SP035171-11	35	mg/kg
SP035171-12	5.2	mg/kg

Opinions and Interpretations: N/A
